# Mold Hysteria: Origin of the Hoax

**DOI:** 10.1080/17402520500131409

**Published:** 2005-06

**Authors:** Christopher Chang, M. Eric Gershwin

**Affiliations:** Division of Rheumatology, Allergy and Clinical Immunology University of California at Davis School of Medicine 451 E. Health Sciences Drive Suite 6510 Davis CA 95616 USA

## Abstract

The topic of building related illness came into the public's eye as a major health issue in the mid 1970s, when several cases of pneumonia were found to be associated with an infectious agent in Philadelphia. This agent was subsequently found to be a gram-positive bacterium known as *Legionella pneumoniae*. During the ensuing 30 years, a myriad of symptom constellations, disorders, clinical syndromes and illnesses have been attributed to indoor living or working environments. Over time, there appeared to be no limit to claims of building related illness, and it was “reported” that almost any kind of clinical symptom, real or imaginary, could be blamed on indoor environments. As society became more and more litigious, many of these disorders were erroneously played out in courtrooms rather than medical offices, creating a circus atmosphere surrounding this class of disorders. With the advent of the internet, as well as other advances in telecommunications, these issues eventually became part of a media frenzy, and all truths could be thrown out the window as issues became more and more decided upon by emotions and unfounded beliefs, rather than scientific data and logical thinking.


*Clinical & Developmental Immunology*, June 2005; 12(2): 151–158

Taylor & Francis

Taylor & Francis Group

### Mold hysteria: Origin of the hoax

CHRISTOPHER CHANG, & M. ERIC GERSHWIN

*Division of Rheumatology, Allergy and Clinical Immunology, University of California at Davis School of Medicine, 451 E. Health Sciences Drive, Suite 6510, Davis, CA 95616, USA*

**Abstract**

The topic of building related illness came into the public's eye as a major health issue in the mid 1970s, when several cases of pneumonia were found to be associated with an infectious agent in Philadelphia. This agent was subsequently found to be a gram-positive bacterium known as *Legionella pneumoniae*. During the ensuing 30 years, a myriad of symptom constellations, disorders, clinical syndromes and illnesses have been attributed to indoor living or working environments. Over time, there appeared to be no limit to claims of building related illness, and it was "reported" that almost any kind of clinical symptom, real or imaginary, could be blamed on indoor environments. As society became more and more litigious, many of these disorders were erroneously played out in courtrooms rather than medical offices, creating a circus atmosphere surrounding this class of disorders. With the advent of the internet, as well as other advances in telecommunications, these issues eventually became part of a media frenzy, and all truths could be thrown out the window as issues became more and more decided upon by emotions and unfounded beliefs, rather than scientific data and logical thinking.

**Keywords:** *Mold, allergies, asthma, hypersensitivity pneumonitis*

### Introduction

It has long been known that the environment plays a role in human health. Generally, environmentally induced adverse effects fall into several broad categories, including allergic, infectious, toxic and psychogenic. The causes of these types of reactions range from living organisms such as bacteria, viruses and fungi and their byproducts, to non-living substances including inorganic, organic and bio-chemicals such as volatile organic chemicals (VOCs), poisons and irritants (Chang and Gershwin 2004, O'Mahony et al. 1989). Examples of these agents are shown in Table I. In fact, as far back as the sixteenth century, indoor living environments have been suspected of causing illness. The Archbishop of Saint Andrews, John Hamilton, suffering from a multitude of respiratory symptoms, was diagnosed by the Italian physician Gerolamo Cardano as being "allergic" to feathers (Chang and Gershwin 2004). Removal of the feathers appeared to solve the problem.

Several factors have contributed to the increased attention focused on building related illness. The very fact that allergies and asthma have seen a marked increase in prevalence over the past thirty years has led to a heightened awareness of all forms of allergic disease as real illnesses. In addition, this increase has been observed to occur mostly in developed countries and in urban areas of developed countries in particular. This coincides with a change in living style in the population of developed countries, partly as a result of building construction standards; from an open air type of environment (e.g. farms), to an energy efficient, tight building type of environment, in which many of us spend up to 90% of our time. Along with our propensity to seek out targets to blame for our misfortune, this led to a fear-induced hysteria which eventually came to be known as the Sick Building Syndrome (Chang et al. 1993).

Correspondence: M. Eric Gershwin, Division of Rheumatology, Allergy and Clinical Immunology, University of California at Davis School of Medicine, 451 E. Health Sciences Drive, Suite 6510, Davis, CA 95616, USA. Tel: 1 530 752 2884. Fax: 1 530 752 4669. E-mail: megershwin@ucdavis.edu

ISSN 1740-2522 print/ISSN 1740-2530 online © 2005 Taylor & Francis Group Ltd
DOI: 10.1080/17402520500131409

Table I. Environmental agents associated with adverse health effects in humans.

| |
|---|
| *Allergenic agents* |
| Non-mold indoor allergens |
| Dust mite |
| Animal dander—cat, dog, rodent, other household pets |
| Cockroach |
| Outdoor allergens |
| Pollens |
| Molds |
| Insects |
| Drugs |
| *Infectious agents* |
| Fungal |
| *A. fumigatus* |
| Allergic fungal sinusitis |
| Viral |
| Bacterial |
| Pneumocystic carinii |
| *Toxins* |
| Gases |
| $SO_2$ |
| $NO_2$ |
| Formaldehyde |
| Radon |
| Volatile organic compounds |
| Particulates |
| Environmental tobacco smoke |
| Asbestos |

### *The Sick Building Syndrome*

The Sick Building Syndrome is a poorly defined clinical entity that is used to describe a constellation of complaints that occur in groups of office workers, in which the complaints are attributed to being present in the building (Chang et al. 1993, Ueno et al. 1986, Chang et al. 1994, Mahmoudi and Gershwin 2000, Assoulin-Daya et al. 2002, Tsai and Gershwin 2002). The symptoms described are numerous, and are frequently different from person to person. These symptoms have included upper and lower respiratory complaints such as difficulty breathing, wheezing, coughing, nasal drainage and congestion, sneezing and tightness in the chest, ocular symptoms such as red, itchy or watery eyes, and a multitude of neurological complaints ranging from headaches to difficulty concentrating and memory loss. Other vague systemic symptoms include arthralgias, fatigue and malaise. Both non-living and living entities have been blamed in Sick Building Syndrome; these include VOC, bacterial, viral or fungal organisms and their byproducts, temperature, vibration, electrostatic fields, lighting and environmental tobacco smoke.

### *Toxic mold syndrome*

In the 1980s attention that was originally focused on Sick Building Syndrome was gradually diverted toward another form of building related illness, a new disease entity called "toxic mold syndrome". Toxic mold syndrome caught the attention of the media and the public after an association between black mold accumulation and perceived occupant symptoms was observed (Nordness et al. 2003). The visible mold in question turned out to be a fungi belonging to the genus *Stachybotrys*, which is a mold that causes a black colored build up of spores on various substrates. Whether or not there is truly a causal relationship between the two is not yet known, as there are currently no studies proving that toxins released by black mold cause the constellation of symptoms commonly described by patients claiming to have "toxic mold syndrome" (Chapman et al. 2003). In fact, it is far more likely that an allergic or immunologic mechanism may be at least partially responsible for symptoms related to exposure to *Stachybotrys* (Edmondson et al. 2005). In no cases is the data convincing. The data is filled with exaggerations and half-truths.

### *The truth about mold*

The relationship between mold exposure and human health has been a prevalent issue in recent decades, and it has been one in which various industries and professions have been involved, including the legal, healthcare, home construction and heating and air-conditioning (HVAC) industries. Like other extraneous living organisms, such as bacteria and viruses, fungi can indeed cause a variety of illnesses ranging from infectious diseases to allergies. Fungi may also be responsible for a number of occupational illnesses known as "hypersensitivity pneumonitis" (Greenberger 2004). It is important to realize that although fungi may cause illness, not all building related illnesses or symptoms can be attributed to fungi simply based on the fact that fungi is present in the environment. Just because something in the environment looks "bad" does not mean that it is necessarily bad for one's health.

The reason this point must be addressed is because many of the judgments in mold related litigation cases are rendered based on appearances and emotion, with no consideration of the scientific evidence available. Often the argument for a causal link between mold and illness is based solely on the unsightly presence of mold. This is analogous to saying that a heap of clothes causes illness because it is untidy. But this argument is exactly what has driven the hype in recent years with regard to "mold madness."

So how does one decipher the mold issue? There are indeed several topics of discussion with regard to mold. For example, what are the real illnesses caused by mold? How do we know this? What are the illnesses that have been attributed to mold, but have not been proven to be a result of mold? What is a "mycotoxin"? Are "mycotoxins" really harmful to human health? Is a mold buildup in the home dangerous to human health? What should one do if one identifies mold in the building? Are there standards as to a safe level of mold in a building? Who cleans up mold-infested buildings? And how do we educate the public in their knowledge of the true data on molds? The remainder of this article will be focused on answering these questions.

*Diseases associated with mold exposure*

For all the hype and hysteria associated with mold related disease, the majority of the cases with the highest publicity are neither mold related nor even real diseases (Terr 2004). Over the past decade, the truth regarding the adverse health effects of molds has become severely distorted by the special interests. In most circles of discussion, when mold is mentioned in the context of human health, the first thing that comes to mind are commonly encountered phrases such as "toxic mold syndrome", or "black mold". The next thing that people think about is that there is someone to blame for the subject's symptomatology, someone that can be held legally accountable, such as a landlord or a builder. In actuality, mold related diseases have only been proven to occur by way of two mechanisms: infectious or allergic. Of the two, allergic diseases as a result of mold exposure are by far the most prevalent. Allergic diseases that can occur as a result of mold exposure are primarily respiratory in nature, leading to allergic rhinitis, conjunctivitis or asthma. In order for mold spores to cause respiratory symptoms, the mold must be airborne. Thus, contrary to popular belief, the presence of mold on a wall has by itself no consistent correlation with health effects.

Indeed, molds are only one category of allergenic entities that can cause allergy or asthma symptoms. Other common indoor allergens that are encountered in indoor environments are shown in Table II, along with their respective sensitization rates (Gruchalla et al. 2005). These allergens are generally carried on particles that are too small (usually less than 10 μm in diameter) to be seen with the naked eye, and it is only when those particles are airborne that they present a human health problem.

Table II. Allergens.

| Allergen | Sensitization rates(14)* (%) |
|---|---|
| Dust mite | 62 |
| Cockroach | 69 |
| Animals | |
| Cat | 44 |
| Dog | 21 |
| Molds | 50 |
| Pollens—tree, weed and grasses | |

*Of 936 children with moderate to severe asthma enrolled in the Inner City Asthma Study Group.

Scientific studies have confirmed that molds can indeed cause allergies, and a mouse model of lung allergy induced by spores of *Cladosporium* and *Alternaria* species was recently developed. *Cladosporium herbarum* and *Alternaria alternata* spores were shown to be able to induce the production of specific IgM and IgG1 antibodies, as well as increasing serum IgE levels. Mold spore challenge also was demonstrated to induce airway hyperreactivity in response to methacholine challenge (Havaux et al. 2005). Obviously, if patients are not allergic (with IgE antibodies) to mold, then they cannot develop such symptoms.

In contrast to allergen related diseases, the entity known as "toxic mold syndrome" is poorly defined clinically, and no mechanism for the pathogenesis of this disorder has been demonstrated. The symptoms that make up this disorder vary from patient to patient and may include respiratory symptoms, headache, mucous membrane irritation, loss of memory, difficulty concentrating, blurry vision, other neurological complaints and skin problems. The etiologic entity for "toxic mold syndrome" is a group of chemicals known as "mycotoxins". The term is derived from "myco," meaning fungal and "toxikon," meaning poison. A practical definition of the term "mycotoxin" is a natural product released by fungi that can evoke a toxic response when presented via a natural route to other living organisms, in particular, higher vertebrates (Bennett 1987). Examples of mycotoxins are shown in Table III. Not all mold species release mycotoxins. Examples of fungi that do produce mycotoxins include *Aspergillus*, *Stachybotrys*, *Fusarium*, *Penicillium* and *Acrimonium* (Jarvis and Miller 2005). Mycotoxins have been shown to be contaminants of grain and other food products, but unless eaten will not make people ill (Robbins et al. 2000; Kelman et al. 2004).

Mycotoxins have been studied *in vitro* in cellular systems, and have been shown to have a number of undesired effects on *in vitro* immune function. EL-4 thymoma cells were found to produce elevated levels of interleukin-2 (IL-2) when cultured in the presence of low levels of satratoxin H, isosatratoxin F, roridin A, and verrucarin A (Lee et al. 1999). On the other hand, the level of IL-2 production was reduced at high concentrations of these mycotoxins. Also observed was a cytotoxic effect that was mycotoxin dosage dependent.

Table III. Mycotoxins.

| |
|---|
| Deoxynivalenol (DON) |
| Diacetoxyscirpenol |
| Nivalenol |
| Satratoxins |
| T-2 toxin |
| Trichoverrins |
| Trichoverrols |
| Verrucarins |
| Verrucarol |

Other mycotoxins have also been found to inhibit protein and DNA synthesis (Ueno et al. 1968, Ueno and Fukushima 1968, Ehrlich and Daigle 1987), cause bone marrow suppression in mice (Ryu et al. 1987) and induce apoptosis of human leukemia cells (Nagase et al. 2001). Mycotoxins have also been found to reduce production of lung surfactant by rabbit type II alveolar cells (Mason et al. 2001). Despite these studies, no adverse effects whatsoever on lung function have been demonstrated to occur as a result of exposure to mycotoxins.

One well-defined entity that was associated with exposure to *Stachybotrys* (black mold) was infant pulmonary hemorrhage. This association first surfaced in the Cleveland, Ohio area in 1994 (Etzel 2003, Etzel et al. 1998, Update 1997). Ten infants presented with pulmonary hemorrhage, with half of them developing recurrent illness. While exposure to *Stachybotrys* was initially thought to be the culprit, this was never proven in subsequent studies. The association was also not reproducible in later studies (Kuhn and Ghannoum 2003, Terr 2001), and the existence of a cause and effect relationship was further refuted by the fact that, while *Stachybotrys* is widespread, infant pulmonary hemorrhage is very rare.

As in the above case, most reports in the literature regarding mold related illnesses are anecdotal and often not accurate. In the case of toxic mold syndrome, studies have failed to prove a relationship (Hardin et al. 2003).

### *Infectious diseases resulting from mold exposure*

Fungi can also cause infectious diseases, including allergic bronchopulmonary aspergillosis (ABPA) (Coop et al. 2004, Malde and Greenberger 2004, Slavin et al. 2004, Wark 2004), allergic fungal sinusitis (Luong and Marple 2004), humidifier fever (Pal et al. 1997, Ohnishi et al. 2002), and a group of conditions known as hypersensitivity pneumonitis (Nacar et al. 2004, Marras et al. 2005, Moore et al. 2005). The fungus most commonly blamed for ABPA is *Aspergillus fumigatus*. The diagnosis can be supported by finding an elevated level of specific IgE to *A. fumigatus*, skin test positivity to *A. fumigatus*, and by direct culture. In contrast, no fungal species has been causally linked to humidifier fever. Both humidifier fever and hypersensitivity pneumonitis present with respiratory symptoms of chest tightness and dyspnea, and other flu-like symptoms such as fever, chills and malaise. Hypersensitivity pneumonitis is associated with occupational asthma, and is described in patients exposed to organic dust. Farmer's lung, pigeon breeder's disease and mushroom worker's disease are examples of hypersensitivity pneumonitis. Although fungi such as *Micropolyspora faenia* and *Actinomycetes* species have been blamed for hypersensitivity pneumonitis, no definitive causal relationship has been found.

### *Non-health related mold hazards*

Diseases proven to be caused by mold include allergies and asthma. But the fact that we have no proof of toxic mold syndrome or infant pulmonary hemorrhage being caused by mold does not mean that mold buildup in homes is harmless. Visible mold may be an indicator of water intrusion into the building, which can damage walls and create structural instability. Moreover, still water collections can also be a medium for the growth of other organisms and may even be a harbor for disease carrying insects like mosquitoes. Increased humidity can also lead to an increase in dust mite exposure, and allergies and asthma symptoms may worsen. Cockroaches also need moisture to survive, and cockroaches have been found to be major contributors to allergy and asthma symptoms, particularly in high-density urban environments. Cockroaches are an indication of poor hygienic conditions, and also poor upkeep of the building. It may eventually be shown that the presence of mold may serve more as an indicator of conditions that are conducive to increasing the risk of exposure to other allergens or infectious agents, rather than itself being harmful to human health.

### *Sampling of mold*

There are generally three ways to sample for mold in indoor environments. The first way is to measure airborne levels of mold. This is the most accurate reflection of exposure, because most mold allergies are mediated via a respiratory route. The symptoms experienced by patients suffering from airborne allergies usually result from inhalation of airborne particles containing these allergens, and include nasal and ocular pruritis, sneezing, rhinitis and nasal congestion, ocular erythema, as well as lower respiratory tract symptoms in asthmatics, such as coughing, wheezing and shortness of breath. Techniques have been developed to collect mold samples from both airborne and surface sources. Airborne samples are collected by using a vacuum pump attached to a compartment holding a collection surface on which particles are deposited, such as a microscope slide for the collection and analysis of non-viable plus viable mold spores, or culture plates for the collection of viable spores only. An example of such devices include the Burkhard sampler, which is a self driven unit consisting of both a vacuum pump and an analysis chamber; another is a simple GAST pump connected to an Anderson sampler. Sampling rates differ for collection of viable versus non-viable samples (15 l/min for viable and 28.6 l/min for non-viable). The pumps are usually run for 3–5 min. Direct visualization of the microscope slide can differentiate mold spores down to the genus, and further speciation can be done using culture techniques. Surface samples are collected using either a tape lift or a swab sample and are more useful for qualitative analysis of the type of mold species present, or for speciation. The third way to analyze mold in the environment is by taking a bulk sample from carpet dust using a standard vacuum cleaner. In the case of *Aspergillus* or *Alternaria*, enzyme linked immunosorbent assays (ELISAs) can be used for quantification of the amount present in bulk samples, as when analyzing other non-mold allergens.

### *A meaningful assessment of indoor mold levels*

In general, indoor levels of mold should reflect outdoor levels. The source of most indoor mold is ultimately what is present outdoors. Outdoor airborne levels of molds can vary significantly based on climate, temperature and relative humidity, foliage, the presence of environmental substrates for mold growth, etc. But whatever the concentration of spores outdoors, the indoor environment presents no additional health hazard to an occupant if the indoor levels are less than or equal to the outdoor levels. It is therefore meaningful, at least when addressing allergies and asthma resulting from molds, to use an indoor/outdoor ratio when evaluating the risk of exposure to indoor mold spores (O'Connor et al. 2004). If indeed the indoor levels of various mold species is different from the outdoor levels, either qualitatively or quantitatively, then one must conclude that there is an alternate source of mold growth. This most often reflects a difference between the indoor and outdoor environments, which usually means that there is an abnormal source of moisture that facilitates mold growth, such as a water leak, or a general increase in humidity. When such a condition is found, then the nature of the difference must be defined. Whether or not such a difference actually is responsible for patient symptoms is yet another matter, as discussed below.

### *Relationship between environmental presence of an allergen and patient symptoms*

Even if mold levels are elevated indoors when compared to outdoor levels, this does not prove that molds are related to an occupant's illness. In the case of allergies, the occupant must exhibit signs that are consistent with allergies, and they must also test positive to the same molds that are found in the environment during RAST or skin testing. If this is not the case, then mold allergies are not responsible for the patient's symptoms. In the case of an infectious disease, there must be a documented infection, such as sinusitis or pneumonia. Such an infection can be documented radiographically, by a complete history and physical and by surgical retrieval of culture samples from the site of infection. In addition, isolation of the organism from the site of infection should also be consistent with what is found in the environment. If this is not found to be the case, then an infectious process due to mold can be ruled out. Vague symptomatology such as headache, malaise, fatigue or difficulty thinking simply is not sufficient to establish a cause and effect relationship. If, after a thorough evaluation of the patient's condition and environment, as illustrated above, the occupant is not allergic to mold, and there is no evidence of a mold infection, nor any evidence that the particular mold in question causes infection, then it is possible that mold is not the issue at all, regardless of what is observed or even what is measured in airborne samples.

### *Issues regarding regulation of indoor air quality and certification of mold specialists*

Recently, many states have begun to set certification standards for mold inspection and remediation specialists. Legislation already exists in New York regarding indoor mold exposure safety limits, and California is in the process of adopting a bill known as the "Toxic Mold Act of 2001", in which standards will be introduced regarding safe indoor levels of mold exposure. Budget considerations have delayed the introduction of such a bill. In Florida, HB117 was introduced and requires that all mold remediation professionals be trained, licensed and certified. While it is admirable that regulations are being proposed to protect individuals from indoor health issues, one would hope that standards are not being arbitrarily introduced based on public hysteria, and that they would be based on evidence and founded on good basic science and clinical studies that are placebo controlled, peer reviewed and reproducible (Anyanwu et al. 2004). Currently, the insurance industry standard is to exclude mold related problems from most home insurance policies. State and federal legislation is being enacted to pass laws regarding mold testing and insurance (Barrett 2003). When purchasing a home or home insurance, it is important for the prospective buyer to be aware of their coverage pertaining to mold-induced health or building problems.

At the present time, given the level of our current knowledge regarding mold related illness, there is no possibility of reaching a consensus on what constitutes a safe absolute level of airborne mold spores (Bobbitt et al. 2005). In fact, if we consider mold exposure as a potential health hazard only from an allergy standpoint, the only number that may be meaningful is the ratio between outdoor and indoor levels.

### *Relating patient symptoms and environmental exposures*

The first step in identifying a building related illness is to evaluate the patient's symptoms. If the patient develops increased symptoms while in the building, then this increases the chance that there is an indoor environmental problem. This in itself does not define the cause of the symptoms, nor does it clarify what type of illness the patient may have. If the symptoms complex is consistent with allergic diseases, then evaluation of the patient for specific allergies and a search for an environmental trigger should be undertaken. If the symptoms are consistent with an infectious disease, the source may be viral, bacterial or fungal, but may or may not be related to occupancy of the building, as infectious symptoms typically do not wax and wane once an exposure has occurred. If symptoms cannot be attributed to a specific condition, or if the symptoms are poorly defined and highly subjective, then there is a high probability that psychosomatic factors may play a role. For example, symptoms such as difficulty concentrating are not typically caused by an allergic mechanism.

Investigation of the environment is an important facet of managing allergic disease. An analysis of the environment for allergens should not be restricted to molds, but should include a search for all of the allergens listed in Table II. The allergenic profile of the patient should be correlated with exposure patterns. If molds are indeed identified as a trigger for the patient's allergies, if the same allergens are found in the environment, and if the indoor mold counts differ significantly from the outdoor mold counts, then a search for an indoor source of the offending mold can be undertaken. An inspection of the building usually identifies this problem. Once the source is found, remediation can ensue.

### *Mold remediation*

There are two separate aspects to mold remediation. The first is health related, and pertains to any allergy or asthma symptoms that may be present in an occupant of a building. If host-environmental relationships fit, then measures should be undertaken to remove the offending mold from the environment. Usually, this can be done by identifying the source of the moisture that is facilitating mold growth. If this is a water leak, repairs may be necessary. Other than that, certain environmental measures can help keep mold levels down, including adjustment of the relative humidity of the home, and removal of potential substrates for mold growth such as damp books, rotting wood, decaying food, etc. Once correction measures have been implemented, airborne mold levels should be re-analyzed. The patient should also be closely monitored by his or her physician to ensure that remedial measures produce the desired improved patient outcomes. Quality of life assessments should be done periodically, and correlated with the patient's environmental exposure. This type of positive feedback seems to provide an additional intangible benefit as it increases patient compliance.

There are several home based programs of environmental avoidance which involve control measures for diminishing exposure to indoor allergens, including the use of mattress encasings, HEPA filters, removal of animals, frequent washing of bed sheets and maintaining hygienic conditions. Results from studies concerning the effectiveness of such home based programs have been extremely promising. When environmental control measures are utilized, it has been demonstrated that allergen loads can be significantly decreased, and an improvement in asthma symptoms has also been shown to occur (Morgan et al. 2004, Crain et al. 2002).

### *Summary*

So what is the "origin" of the hoax? What created and fueled the existence of the past two decades of "mold

Table IV. A sampling of mold-litigation cases.

| Year | Case | Issue | Disposition |
|---|---|---|---|
| 2001 | Ballard vs. Fire Insurance Exchange | *Stachybotrys* and mycotoxins and disclosure issues | Jury verdict in favor of plaintiffs for $32 million, including $12 million in punitive damages and $8.9 million in legal fees |
| 1999 | MacDonald vs. Dufferin-Peel Catholic District School Board | Exposure to toxic mold resulted in ailments | Seeking $1 billion in general damages, $500 million in special damage costs and an additional $500 million in damages to parents |
| 2001 | Erin Brockovich vs. Robert Selleck | Water intrusion led to growth of mold, adverse health effects from the exposure, Brockovich sued former owner Selleck | Symptoms probably a result of indoor pet allergens (Brockovich's own dog) |
| 1999 | New Haverford Partnership, et al. vs. Elizabeth Stroot | Exposure to various mycotoxins | Jury awarded $1 million in damages to Stroot; verdict upheld on appeal |
| 1997 | Doe Homeowners vs. Roe Seller | Toxic mold caused bodily injury and property damage | Case settled for $1.3 million |

hysteria?" In all probability, there was not one single reason why this has mushroomed so out of control. We live in a very litigious society, and we are quick to apply blame to others, regardless of guilt. In addition, many of us live in fear of one thing or another, and issues tend to be sensationalized based on this fear. In this case, the fear is that something "ugly" (i.e. mold growth) is going to harm us in some way. Suffice to say that mold hysteria did not arise out of scientific knowledge or clinical studies. Even to this day, court judgments, jury decisions and awards are based on emotion and junk science (Lees-Haley 2003). Literally thousand of mold cases have been brought forth, and heavy settlements and judgments have been awarded, the only commonality of which is the conspicuous absence of any scientific evidence. A sample of landmark mold litigation cases is illustrated in Table IV. The entire current situation regarding mold-related illness is unfortunate as it detracts from the truth about mold.

### References

Anyanwu EC, Campbell AW, Ehiri JE. 2004. Mycotoxins and antifungal drug interactions: Implications in the treatment of illnesses due to indoor chronic toxigenic mold exposures. Scientific World J 4:167–177.

Assoulin-Daya Y, Leong A, Shoenfeld Y, Gershwin ME. 2002. Studies of sick building syndrome. IV. Mycotoxicosis. J Asthma 39(3):191–201.

Barrett JR. 2003. Mold insurance: Crafting coverage for a spreading problem. Environ Health Perspect 111(2):A100–A103.

Bennett JW. 1987. Mycotoxins, mycotoxicoses, mycotoxicology and mycopathologia. Mycopathologia 100(1):3–5.

Bobbitt Jr, RC, Crandall MS, Venkataraman A, Bernstein JA. 2005. Characterization of a population presenting with suspected mold-related health effects. Ann Allergy Asthma Immunol 94(1):39–44.

Chang C, Gershwin ME. 2004. Indoor air quality and human health: Truth vs mass hysteria. Clin Rev Allergy Immunol 27(3):219–240.

Chang CC, Ruhl RA, Halpern GM, Gershwin ME. 1993. The sick building syndrome. I. Definition and epidemiological considerations. J Asthma 30(4):285–295.

Chang CC, Ruhl RA, Halpern GM, Gershwin ME. 1994. Building components contributors of the sick building syndrome. J Asthma 31(2):127–137.

Chapman JA, Terr AI, Jacobs RL, Charlesworth EN, Bardana Jr, EJ. 2003. Toxic mold: Phantom risk vs science. Ann Allergy Asthma Immunol 91(3):222–232.

Coop C, England RW, Quinn JM. 2004. Allergic bronchopulmonary aspergillosis masquerading as invasive pulmonary aspergillosis. Allergy Asthma Proc. 25(4):263–266.

Crain EF, Walter M, O'Connor GT, et al. 2002. Home and allergic characteristics of children with asthma in seven U.S. urban communities and design of an environmental intervention: The Inner-City Asthma Study. Environ Health Perspect 110(9):939–945.

Edmondson DA, Nordness ME, Zacharisen MC, Kurup VP, Fink JN. 2005. Allergy and "toxic mold syndrome". Ann Allergy Asthma Immunol 94(2):234–239.

Ehrlich KC, Daigle KW. 1987. Protein synthesis inhibition by 8-oxo-12,13-epoxytrichothecenes. Biochim Biophys Acta 923(2):206–213.

Etzel RA. 2003. Stachybotrys. Curr Opin Pediatr 15(1): 103–106.

Etzel RA, Montana E, Sorenson WG, et al. 1998. Acute pulmonary hemorrhage in infants associated with exposure to Stachybotrys atra and other fungi. Arch Pediatr Adolesc Med 152(8):757–762.

Greenberger PA. 2004. Mold-induced hypersensitivity pneumonitis. Allergy Asthma Proc 25(4):219–223.

Gruchalla RS, Pongracic J, Plaut M, et al. 2005. Inner city asthma study: Relationships among sensitivity, allergen exposure, and asthma morbidity. J Allergy Clin Immunol 115(3):478–485.

Hardin BD, Kelman BJ, Saxon A. 2003. Adverse human health effects associated with molds in the indoor environment. J Occup Environ Med 45(5):470–478.

Havaux X, Zeine A, Dits A, Denis O. 2005. A new mouse model of lung allergy induced by the spores of *Alternaria alternata* and *Cladosporium herbarum* molds. Clin Exp Immunol 139(2):179–188.

Jarvis BB, Miller JD. 2005. Mycotoxins as harmful indoor air contaminants. Appl Microbiol Biotechnol 66(4):367–372.

Kelman BJ, Robbins CA, Swenson LJ, Hardin BD. 2004. Risk from inhaled mycotoxins in indoor office and residential environments. Int J Toxicol 23(1):3–10.

Kuhn DM, Ghannoum MA. 2003. Indoor mold, toxigenic fungi, and Stachybotrys chartarum: Infectious disease perspective. Clin Microbiol Rev 16(1):144–172.

Lee MG, Li S, Jarvis BB, Pestka JJ. 1999. Effects of satratoxins and other macrocyclic trichothecenes on IL-2 production and viability of EL-4 thymoma cells. J Toxicol Environ Health A 57(7):459–474.

Lees-Haley PR. 2003. Toxic mold and mycotoxins in neurotoxicity cases: *Stachybotrys*, *Fusarium*, *Trichoderma*, *Aspergillus*, *Penicillium*, *Cladosporium*, *Alternaria*, *Trichothecenes*. Psychol Rep 93(2):561–584.

Luong A, Marple BF. 2004. Allergic fungal rhinosinusitis. Curr Allergy Asthma Rep 4(6):465–470.

Mahmoudi M, Gershwin ME. 2000. Sick building syndrome. III. *Stachybotrys chartarum*. J Asthma 37(2):191–198.

Malde B, Greenberger PA. 2004. Allergic bronchopulmonary aspergillosis. Allergy Asthma Proc 25(4 Suppl. 1):S38–S39.

Marras TK, Wallace Jr. RJ, Koth LL, Stulbarg MS, Cowl CT, Daley CL. 2005. Hypersensitivity pneumonitis reaction to *Mycobacterium avium* in household water. Chest 127(2):664–671.

Mason CD, Rand TG, Oulton M, MacDonald J, Anthes M. 2001. Effects of *Stachybotrys chartarum* on surfactant convertase activity in juvenile mice. Toxicol Appl Pharmacol 172(1):21–28.

Moore JE, Convery RP, Millar BC, Rao JR, Elborn JS. 2005. Hypersensitivity pneumonitis associated with mushroom worker's lung: An update on the clinical significance of the importation of exotic mushroom varieties. Int Arch Allergy Immunol 136(1):98–102.

Morgan WJ, Crain EF, Gruchalla RS, et al. 2004. Results of a home-based environmental intervention among urban children with asthma. N Engl J Med 351(11):1068–1080.

Nacar N, Kiper N, Yalcin E, et al. 2004. Hypersensitivity pneumonitis in children: Pigeon breeder's disease. Ann Trop Paediatr 24(4):349–355.

Nagase M, Alam MM, Tsushima A, Yoshizawa T, Sakato N. 2001. Apoptosis induction by T-2 toxin: Activation of caspase-9, caspase-3, and DFF-40/CAD through cytosolic release of cytochrome c in HL-60 cells. Biosci Biotechnol Biochem 65(8):1741–1747.

Nordness ME, Zacharisen MC, Fink JN. 2003. Toxic and other non-IgE-mediated effects of fungal exposures. Curr Allergy Asthma Rep 3(5):438–446.

O'Connor GT, Walter M, Mitchell H, et al. 2004. Airborne fungi in the homes of children with asthma in low-income urban communities: The Inner-City Asthma Study. J Allergy Clin Immunol 114(3):599–606.

Ohnishi H, Yokoyama A, Hamada H, et al. 2002. Humidifier lung: Possible contribution of endotoxin-induced lung injury. Intern Med 41(12):1179–1182.

O'Mahony M, Lakhani A, Stephens A, Wallace JG, Youngs ER, Harper D. 1989. Legionnaires' disease and the sick-building syndrome. Epidemiol Infect 103(2):285–292.

Pal TM, de Monchy JG, Groothoff JW, Post D. 1997. The clinical spectrum of humidifier disease in synthetic fiber plants. Am J Ind Med 31(6):682–692.

Robbins CA, Swenson LJ, Nealley ML, Gots RE, Kelman BJ. 2000. Health effects of mycotoxins in indoor air: A critical review. Appl Occup Environ Hyg 15(10):773–784.

Ryu JC, Ohtsubo K, Izumiyama N, Mori M, Tanaka T, Ueno Y. 1987. Effects of nivalenol on the bone marrow in mice. J Toxicol Sci 12(1):11–21.

Slavin RG, Hutcheson PS, Chauhan B, Bellone CJ. 2004. An overview of allergic bronchopulmonary aspergillosis with some new insights. Allergy Asthma Proc 25(6):395–399.

Terr AI. 2001. *Stachybotrys*: Relevance to human disease. Ann Allergy Asthma Immunol 87(6 Suppl. 3):57–63.

Terr AI. 2004. Are indoor molds causing a new disease? J Allergy Clin Immunol 113(2):221–226.

Tsai YJ, Gershwin ME. 2002. The sick building syndrome: What is it when it is? Compr Ther 28(2):140–144.

Ueno Y, Fukushima K. 1968. Inhibition of protein and DNA syntheses in Ehrlich ascites tumour by nivalenol, a toxic principle of Fusarium nivale-growing rice. Experientia 24(10):1032–1033.

Ueno Y, Hosoya M, Morita Y, Ueno I, Tatsuno T. 1968. Inhibition of the protein synthesis in rabbit reticulocyte by nivalenol, a toxic principle isolated from Fusarium nivale-growing rice. J Biochem (Tokyo) 64(4):479–485.

Ueno Y, Lee US, Tanaka T, Hasegawa A, Matsuki Y. 1986. Examination of Chinese and U.S.S.R. cereals for the Fusarium mycotoxins, nivalenol, deoxynivalenol and zearalenone. Toxicon 24(6):618–621.

1997. Update: Pulmonary hemorrhage/hemosiderosis among infants—Cleveland, Ohio, 1993–1996. MMWR Morb Mortal Wkly Rep 46(2):33–35.

Wark P. 2004. Pathogenesis of allergic bronchopulmonary aspergillosis and an evidence-based review of azoles in treatment. Respir Med 98(10):915–923.

